# Developing an international *Pseudomonas aeruginosa* reference panel

**DOI:** 10.1002/mbo3.141

**Published:** 2013-11-11

**Authors:** Anthony De Soyza, Amanda J Hall, Eshwar Mahenthiralingam, Pavel Drevinek, Wieslaw Kaca, Zuzanna Drulis-Kawa, Stoyanka R Stoitsova, Veronika Toth, Tom Coenye, James E A Zlosnik, Jane L Burns, Isabel Sá-Correia, Daniel De Vos, Jean-Paul Pirnay, Timothy J Kidd, David Reid, Jim Manos, Jens Klockgether, Lutz Wiehlmann, Burkhard Tümmler, Siobhán McClean, Craig Winstanley, on behalf of EU FP7 funded COST Action BM1003 “Cell surface virulence determinants of cystic fibrosis pathogens”

**Affiliations:** 1 Institute of Cellular Medicine, Newcastle UniversityNewcastle, U.K; 2 Institute of Infection & Global Health, University of LiverpoolLiverpool, U.K; 3 Organisms and Environment Division, Cardiff School of Biosciences, Cardiff UniversityCardiff, U.K; 4 Department of Medical Microbiology, 2nd Faculty of Medicine, Charles University and University Hospital MotolPrague, Czech Republic; 5 The Jan Kochanowski UniversityKielce, Poland; 6 Institute of Genetics and Microbiology, University of WroclawWroclaw, Poland; 7 The Stephan Angeloff Institute of MicrobiologySofia, Bulgaria; 8 Semmelweis UniversityBudapest, Hungary; 9 Universiteit GentGent, Belgium; 10 Centre for Understanding and Preventing Infection in Children, Department of Pediatrics, Faculty of Medicine, University of British ColumbiaVancouver, Canada; 11 Seattle Children's HospitalSeattle, Washington; 12 IBB (Institute for Biotechnology and Bioengineering), CEBQ, Instituto SuperiorTécnico, Universidade de LisboaLisboa, Portugal; 13 Queen Astrid Military HospitalBrussels, Belgium; 14 Queensland Children's Medical Research Institute, The University of QueenslandBrisbane, Queensland, Australia; 15 The Prince Charles HospitalBrisbane, Queensland, Australia; 16 Department of Infectious Diseases and Immunology, Sydney Medical School, University of SydneySydney, Australia; 17 Hannover Medical SchoolHannover, Germany; 18 Centre of Microbial Host Interactions, Institute of TechnologyTallaght, Ireland

**Keywords:** Cystic fibrosis, genotype, pathogen, *Pseudomonas aeruginosa*

## Abstract

*Pseudomonas aeruginosa* is a major opportunistic pathogen in cystic fibrosis (CF) patients and causes a wide range of infections among other susceptible populations. Its inherent resistance to many antimicrobials also makes it difficult to treat infections with this pathogen. Recent evidence has highlighted the diversity of this species, yet despite this, the majority of studies on virulence and pathogenesis focus on a small number of strains. There is a pressing need for a *P. aeruginosa* reference panel to harmonize and coordinate the collective efforts of the *P. aeruginosa* research community. We have collated a panel of 43 *P. aeruginosa* strains that reflects the organism's diversity. In addition to the commonly studied clones, this panel includes transmissible strains, sequential CF isolates, strains with specific virulence characteristics, and strains that represent serotype, genotype or geographic diversity. This focussed panel of *P. aeruginosa* isolates will help accelerate and consolidate the discovery of virulence determinants, improve our understanding of the pathogenesis of infections caused by this pathogen, and provide the community with a valuable resource for the testing of novel therapeutic agents.

## Background

Cystic fibrosis (CF) is a significant health care challenge and an important cause of premature mortality. Chronic lower respiratory tract infections are the major cause of morbidity and mortality in CF. Impaired mucociliary clearance from the lung makes CF patients vulnerable to opportunistic infections. Novel data from nonculture-based techniques suggests that the airway microbiome in CF is polymicrobial with multiple organisms present (Blainey et al. 2012; Fodor et al. 2012; Zhao et al. 2012). Despite these methodologic advances, conventional culture techniques remain the main clinical tool used in managing CF infections. Such conventional culture methods reveal that a relatively limited number of pathogens are isolated during pulmonary infections seen in patients with CF. The pathogens isolated by culture are predominantly *Staphylococcus aureus*, *Haemophilus influenzae*, and *Pseudomonas aeruginosa*. Rarer organisms from the *Burkholderia cepacia* complex (Bcc) and other pathogens are also encountered (de Soyza et al. 2004; Davies and Rubin 2007). Although Bcc causes less than 10% of CF infections (Lipuma 2010), the established international Bcc reference panel has helped harmonize *Burkholderia* research by standardizing approaches (Mahenthiralingam et al. 2000; Coenye et al. 2003).

*Pseudomonas aeruginosa* is the major pathogen in CF, infecting up to 80% of adult patients, and once established, the pathogen is often difficult to treat clinically (Cheng et al. 1996; Fothergill et al. 2012a; Parkins et al. 2012). Surprisingly there are no recognized international reference panels for the more prevalent CF pathogens, such as *P. aeruginosa*, *S. aureus* or *H. influenzae*. Arguably the most pressing need is for an international *P. aeruginosa* reference panel to reflect the relevance of this pathogen to CF and a range of other infections. Such a panel will encourage researchers to avoid use of isolates with limited availability, and to potentially prevent unnecessary repetition across laboratories. The availability of a standardized reference panel would improve efficiency and reduce experimental animal sacrifice, while also facilitating the search for improved therapeutic approaches. In order to assemble the most appropriate reference panel of *P. aeruginosa* isolates, we aimed to define consensus on the core characteristics of an international reference panel through an iterative and interactive process involving workshops and a consensus finding exercise. Molecular genotyping was then used to ensure that the panel was broadly representative of the wider population structure of *P. aeruginosa*.

## Methods

### Consensus choice of isolates for initial inclusion

A broad range of expertise was assembled including clinicians, clinical microbiologists and basic science microbiology researchers. Requirements for a reference panel were discussed in open forum on two occasions under the auspices of a European Union Co-ordinated Scientific and Technology (COST) action (COST BM1003; http://www.cost-bm1003.info/). Discussion with further researchers active in the field with an international perspective (including coauthors D. D. V., J. P. P., T. K., J. B., and B. T.; see also acknowledgments) was then conducted prior to a final consensus process involving COST action members using prior techniques (RAND consensus tool) (Francis et al. 2007).

The consensus-seeking process used statements identified in the prior workshops, with the assembled experts independently scoring each statement. Statements identified and scored in the RAND process are included in Table 1. Consensus was then sought based on the individual scores as compared to the group average. Indifference was rated as scores 4–6, while 7–9 was rated as a positive consensus and 1–3 as a negative consensus. The group were also asked to rate the ideal number of isolates to be included in the panel with a mean score presented.

**Table 1:** Details of the criteria used in the consensus process and the outcome. Please grade the following criteria as regards how necessary they are to necessitate inclusion of a particular *Pseudomonas aeruginosa* strain into an international reference panel (increasing numbers mean increasing necessity): *Consensus grading* – NR, consensus not reached; 9, mandatory criteria; 7, 8, necessary criteria; 4, 5, 6, useful but not mandatory; 2, 3, not a necessary criteria; 1, no relevance to international reference panel.

	Consensus grade
The *Pseudomonas* strain has	
Already been genome sequenced	5
Some prior virulence data in vitro	6
Extensive prior virulence data in vitro	NR
Some prior virulence data in vivo	NR
Extensive prior virulence data in vivo	NR
A known biofilm formation phenotype	NR
Can be easily phenotyped for biofilm phenotype	NR
Comes from a clinical source other than CF	6
Comes only from CF sources	NR
Is known to be from early or late stage CF	NR
Is of mucoid nature	NR
Is of non mucoid nature	NR
Is susceptible to bacteriophages	NR
The strain panel should include	
Range of strains from different sources (geographic)	8
Range of strains from different sources (CF, non CF clinical and environmental)	8
ONLY genome sequenced strains	4
Strains with structural characterization data available	6
ONLY strains where structure of virulence determinants known	NR
Strains where variation in resistance is known or expected	6
ONLY strains where resistance patterns already known	NR
Strains where in vivo virulence patterns where possible	6
ONLY strains where in vivo virulence patterns already known	NR
Strains to allow a variety of Mucoid\ non Mucoid where possible	6
Strains ONLY where non Mucoid \non Mucoid Status known	NR
Strains of epidemic and sporadic clinical infection status	8
The panel should be provided at no cost	NR
The panel should be hosted by a recognized collection (e.g., BCCM)	8
The ideal number of strains in the panel should be 0–10,11–20, 21–30, 31–40, 41–50	21–30

### Genotyping of isolates using the ArrayTube method

Strains of *P. aeruginosa* were genotyped using the ArrayTube (AT) system (CLONDIAG, Alere Technologies, Köln, Germany) as described previously (Wiehlmann et al. 2007). The AT microarray chip enables strains to be classified according to 13 core genome single-nucleotide polymorphisms (SNPs), and also screens for 38 variable genetic markers of the *P. aeruginosa* accessory genome. These include several previously reported genomic islands (Arora et al. 2001; Liang et al. 2001; Larbig et al. 2002; de Chial et al. 2003; Spencer et al. 2003; He et al. 2004; Klockgether et al. 2004; Lee et al. 2006). Data from the 13 SNPs are combined with flagellin type (a/b) and the presence of the genes encoding mutually exclusive type III secretion exotoxins (S or U), to generate a strain-specific “hexadecimal code” represented by four digits (Wiehlmann et al. 2007). This code can be used to search a large database of *P. aeruginosa* strains (Cramer et al. 2012). Subsequently, eBURST (version 3.0) (Feil et al. 2004; Spratt et al. 2004) analysis of data generated using the AT from the 13 SNPs, flagellin type (a or b) and presence of the mutually exclusive type III secretion exotoxins (S or U), was used to visualize the position of panel strains within the wider *P. aeruginosa* population structure using a database of 955 genotyped strains (Cramer et al. 2012).

## Results

### RAND process

A number of strain and reference panel characteristics were identified at the scoping workshops (Table 1). Through the iterative consensus-seeking process we identified a variety of characteristics for individual isolates and also for the overall panel, and then reduced these to criteria that were deemed either mandatory or critical. Consensus was reached where the majority of scores were within the same ranking and was achieved in 12 of 28 statements. The preferred mean number of isolates to be included in the reference panel was 26 ± 4 (10 responders).

### Panel strain selection

Isolates were selected based on the characteristics identified through the Delphi process and literature searches via PubMed. Where possible, isolates were chosen when extensive prior data were available, including those with genome sequencing data (Stover et al. 2000; Mathee et al. 2008; Winstanley et al. 2009; Stewart et al. 2011), in vitro and/or in vivo virulence data (Hajjar et al. 2002; Al-Aloul et al. 2004; Cigana et al. 2009; Carter et al. 2010). Isolates were also selected to include representatives from diverse biologic and geographic origins, including clinical isolates (CF and non-CF clinical infections) and environmental sources (Cigana et al. 2009; Pirnay et al. 2009). Because this is an International panel, a global perspective is essential and isolates from geographically dispersed origins were also chosen (Pirnay et al. 2009). The final selection of 43 strains is listed in Table 2. The strain panel is available from the BCCM/LMG Bacteria Collection (http://bccm.belspo.be/about/lmg.php), Gent, Belgium, and the LMG reference numbers are shown in Table 2.

**Table 2:** International reference panel of *Pseudomonas aeruginosa* isolates.

Panel no.	Source ID	LMG number	Origin and source	Genome sequence	AT code (Clone)	Details	Reference
**CF transmissible strains**							
1	LES B58	27622	CF, U.K. (1988)	Yes	4C12(T)	Earliest isolate of LES; genome sequenced; produces more biofilm than PAO1 and PA14 but less motile; virulent in *C. elegans* model, reduced virulence in murine model	Kukavica-Ibrulj et al. (2008); Winstanley et al. (2009); Carter et al. (2010)
2	LES 400	27623	CF, U.K.	Yes	4C12(T)	Quorum sensing defective *lasR* mutant of LES; reduced *C. elegans* killing; upregulation of alginate production	Salunkhe et al. (2005); Carter et al. (2010)
3	LES 431	27624	Non CF parent of CF patient, U.K.	Yes	4C12(T)	LES isolate with increased virulence in the murine model; hypervirulent subtype more adapted to acute infections; transmitted to non CF parents; upregulated QS genes	Salunkhe et al. (2005); Carter et al. (2010)
4	C3719	27625	CF, Manchester, U.K.	Yes	4012(O)	Manchester epidemic strains associated with increased treatment requirements of CF patients with a highly transmissible strain	Jones et al. (2002); Mathee et al. (2008)
5	DK2	27626	CF, Denmark; earliest isolates 1973–2007	Yes	F421(A2)	Initial period of rapid adaption in CF host, followed by long period of some phenotypic changes and genotypic negative selection; low growth rate, loss of motility and reduced regulatory functions	Yang et al. (2011b)
6	AES-1R	27627	Pediatric CF (1992), Melbourne, Australia	Yes	E82A	AUST-01 strain (aka AES-1, Pulsotype 1), ST-649; associated with increased morbidity and treatment; virulence gene expression during chronic CF infection; niche specialization	Armstrong et al. (2002); Manos et al. (2008); Naughton et al. (2011)
7	AUS23	27628	Adult CF (2007), Brisbane, Australia	Pending	2D9A	AUST-02 strain (aka AES-2, Pulsotype 2), ST-775; associated with increased treatment requirements and niche specialization	O'Carroll et al. (2004); Kidd et al. (2012)
8	AUS52	27629	Adult CF (2008), Hobart, Australia	Pending	EC22	AUST-03 strain (aka AES-3), ST-242; associated with increased exacerbation rate; predominantly detected in CF patients from Tasmania and southern Australia	Bradbury et al. (2008); Kidd et al. (2012)
**Paired or sequential CF isolates**							
9	AA2	27630	CF (early), Germany	Yes	AF9A	LPS and PGN studied; in vivo virulence	Bragonzi et al. (2006, 2009); Lore et al. (2012)
10	AA43	27631	CF (late), Germany	Yes	AF9A	As above	Bragonzi et al. (2006, 2009); Lore et al. (2012)
11	AA44	27632	CF (late), Germany	Yes	AF9A	As above	(Bragonzi et al. 2006, 2009;Lore et al. 2012)
12	AMT 0023-30	27633	Pediatric CF, Seattle, WA	Yes	478A	Early isolate taken at 6 months of age; persister cells	Mulcahy et al. (2010)
13	AMT 0023-34	27634	Pediatric CF, Seattle, WA	Yes	478A	96-month isolate from same patient as AMT023-30 showing a 100-fold increase in persister cells; *mutS* mutation; hypermutator phenotype	Mulcahy et al. (2010)
14	AMT 0060-1	27635	Pediatric CF, Seattle, WA	No	6D92(H)	Late isolate identifying *hip* mutants; late isolates showed high-level persister cells; mutation in *mexZ* repressor affecting MIC to Ofloxacin, Carbenicillin and Tobramycin.	Mulcahy et al. (2010)
15	AMT 0060-2	27636	Pediatric CF, Seattle, WA	No	6D92(H)	Late isolate identifying *hip* mutants; late isolates from these patients showed high level persister cells, mutation in *mexZ* repressor affecting MIC to Ofloxacin, Carbenicillin and Tobramycin.	Mulcahy et al. (2010)
16	AMT 0060-3	27637	Pediatric CF, Seattle, WA	Pending	6D92(H)	Early isolate from same patient as AMT 0060-1 and -2 were isolated	Mulcahy et al. (2010)
**Commonly used strains/clones**							
17	PAO1^1^ (ATCC 15692)	27638	Genome sequenced isolate		0002(W)	Widely studied wound isolate from Melbourne, Australia; characterized by Bruce Holloway	Stover et al. (2000)
18	UCBPP-PA14	27639	Human Burn isolate	Yes	D421(A)	Severe symptoms in mouse burn model; 58 genes present in PA14 not in PAO1	Rahme et al. (1995); Lee et al. (2006)
19	PAK	27640	Clinical non CF	Pending	55AA	Widely studied; expresses pili, flagella and glycosylation islands etc.	Totten and Lory (1990); Semblat and Doerig (2012)
20	CHA	27641	CF	Yes	EC2A	Detailed phenotypic characterization;	Toussaint et al. (1993); Dacheux et al. (2000); Bezuidt et al. (2013)
21	NN2	27642	CF, Germany	Yes	C40A (C)	Detailed phenotypic characterization; Clone C	Cramer (et al. 2011)
**Strains with specific phenotypic/virulence characteristics**							
22	IST 27 mucoid	27643	Lisbon Portugal, CF patient	Pending	F69A	Pediatric CF patient from Hospital de Santa Maria,Lisbon	Leitao et al. (1996); Tavares et al. (1999)
23	IST 27N	27644	Lisbon Portugal, spontaneous nonmucoid variant of IST27	Pending	F69A	Spontaneous non-mucoid variant of IST27 with the same macrofragment restriction profile by RFLP-PFGE	Leitao et al. (1996); Tavares et al. (1999)
24	968333S	27645	UK Non CF bronchiectasis	Pending	3C52(K)	Non CF bronchiectasis patient from the UK; Mucoid; Detailed phenotypic characterization	De Soyza et al. (Manuscript under review)^2^
25	679	27646	Non CF Urine sample, male, Wroclaw Poland, 2011	Pending	0C4A	Resistant to imipenem and meropenem	Zuzanna Drulis-Kawa, University of Wroclaw
26	39016	27647	Keratitis eye isolate, U.K.	Yes	F469 (D)	Clone D by AT; serotype O11; carries distinctive *pilA*; subpopulation adapted to corneal infections; associated with severe infection; ST-235	Stewart et al. (2011); Hall et al. (2013)
27	2192	27648	Chronic CF patient, Boston, MA	Yes	049A	Large genome, converted to mucoidy; produces LPS which lacks O-side chains; lacks motility	Mathee et al. (2008)
28	NH57388A	27649	Denmark CF	Pending	6822	Stable CF sputum mucoid isolate; alginate hyperproducer; has functional QS system; caused chronic lung infection in a mouse model.	Hoffmann et al. (2005)
**Strains included to represent serotype, genotype, geographic and source diversity**							
29	1709-12	27650	Leuven Belgium Non CF clinical 2004	Pending	7D92	Serotype 12; multi drug resistant	Pirnay et al. (2009)
30	Mi 162	27651	Non CF burn, Ann Arbor, MI, 1997	Pending	D429(Q)	Serotype 11; multi drug resistant	Pirnay et al. (2009)
31	Jpn 1563	27652	Lake Tamaco, Japan, Lake water, 2003	Pending	3C1A	Non serotypeable	Pirnay et al. (2009)
32	LMG 14084	27653	Bucharest, Romania, Water, 1960-1964	Pending	E42A (B)	Serotype 17	Pirnay et al. (2009)
33	Pr335	27654	Prague, Czech Republic, Hospital environment 1997	Pending	FA0A	Serotype 1	Pirnay et al. (2009)
34	U018a	27655	Hobart, Australia, CF patient	Pending	2A42	Serotype 1	Pirnay et al. (2009)
35	CPHL 9433	27656	Tobacco plant, Philippines	Pending	F462	Serotype 1	Pirnay et al. (2009)
36	RP1	27657	CF, Germany	Yes	OC2E	Abundant in Northern Germany but infrequent in other European countries	Cramer et al. (2012)
37	15108/-1	27658	ICU (acute infection), Spain	Yes	E429	Isolated in Prat de Llobgregat, Spain; Detailed phenotypic characterization	Kohler et al. (2009)
38	57P31PA	27659	Chronic Obstructive Pulmonary Disease, USA	Yes	2C22	Detailed phenotypic characterization	Rakhimova et al. (2009)
39	13121/-1	27660	ICU (acute infection), France	Yes	239A	Isolated inLimoges, France; Detailed phenotypic characterization	Wiehlmann et al. (2007)
40	39177	27661	Keratitis, Manchester U.K.	Yes	EA0A	Serotype 1; Detailed phenotypic characterization	Stewart et al. (2011); (Shankar et al. (2012)
41	KK1	27662	CF, Germany	Yes	1BAE	Detailed phenotypic characterization	Cramer et al. (2012)
42	A5803	27663	Community-acquired pneumonia	Yes	F429	Detailed phenotypic characterization	Wiehlmann et al. (2007)
43	TBCF10839	27664	CF, Germany	Yes	3C52	Detailed phenotypic characterization	Bezuidt et al. (2013); Klockgether et al. (2013)

MIC, minimum inhibitory concentration; ICU, intensive care unit.

1 This has alternate strain names of: 1C, ATCC 15692, ATCC 17503, ATCC 25247, ATCC 25375, BCRC 13078, CCRC 13078, CECT 4122, CIP 104116, HER 1146, Holloway 1C, Holloway1, JCM 14847, KCTC 1637, Kemira Oy, LMG 12228, NCCB 4163, NCIMB 10545, NCIMB 10548, PA0 1, PA01, PAO 1, PC 4163, PRS 101, Stanier 131, VTT E-082794, VTT E-84219.

2 De Soyza, A., A. Perry, A. J. Hall, S. Sunny, K. Walton, N. H. Mustafa, et al. *Pseudomonas aeruginosa* cross infection in bronchiectasis: a Molecular Epidemiology study. Manuscript under review.

### Genotyping to define the distribution of the panel strains among the wider *P. aeruginosa* population

**Figure 1:**
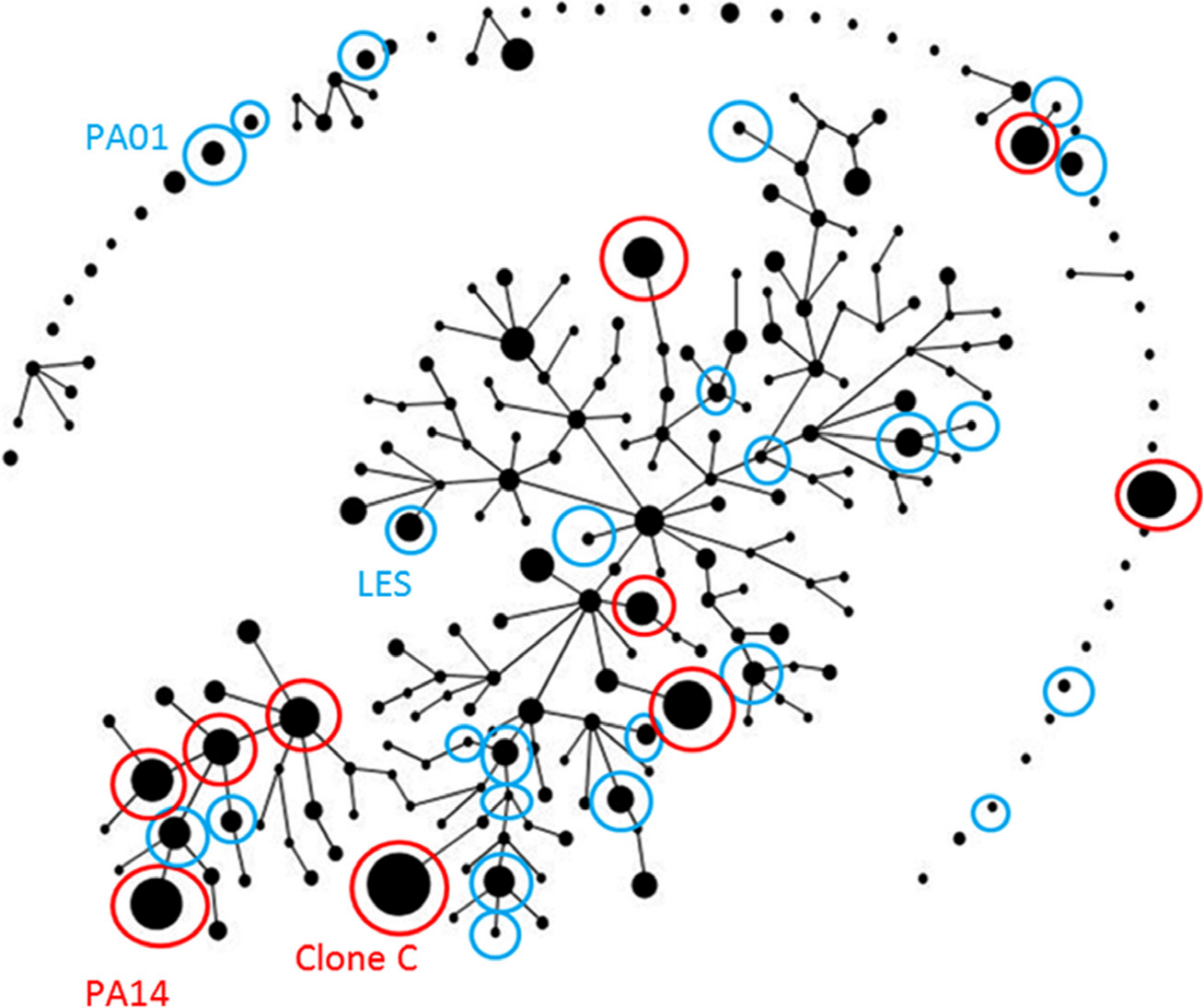
Distribution of panel strains among the wider *Pseudomonas aeruginosa* population. The figure shows an eBURST analysis based on AT genotyping using a database of 955 *P. aeruginosa* isolates of independent origin. The locations of the panel isolates are circled. Red circles indicate genotypes corresponding to the 10 most abundant in the database. Blue circles indicate the location of other panel strains. Each dot represents a different genotype; the size of a dot indicates the relative abundance of a genotype. The genotype cluster locations for four commonly studied strains from the panel are indicated.

Each of the panel strains was genotyped using the AT method. Figure 1 shows an eBURST representation of the distribution of the panel strains among the wider population of *P. aeruginosa*. The panel strains are widely distributed and include representatives of both abundant and less abundant clones. The AT codes are indicated in Table 2.

### Brief description of the panel strains

#### CF transmissible strains

Although it is still widely assumed that most CF patients acquire their infecting strains of *P. aeruginosa* from environmental sources, there is increasing evidence for the emergence of particularly successful transmissible strains (Fothergill et al. 2012a), some of which have been associated with increased patient morbidity or mortality (Al-Aloul et al. 2004; Aaron et al. 2010) or antimicrobial resistance (Ashish et al. 2012). Hence, we have included representatives of the most widely studied transmissible strains. These include three isolates of the Liverpool Epidemic Strain (LES), first reported in a U.K. children's CF Unit in the 1990s (Cheng et al. 1996), but known to be widespread through the U.K. (Scott and Pitt 2004; Martin et al. 2013), and reported recently in North America (Aaron et al. 2010). This strain is associated with novel transmission events (McCallum et al. 2002), increased patient morbidity (Al-Aloul et al. 2004), and resistance to antimicrobials (Ashish et al. 2012). There are many phenotypic variants of this strain (Mowat et al. 2011), but we have selected three, namely (1) LESB58, the earliest known isolate (from 1988) (Winstanley et al. 2009), (2) LES400, a *lasR* mutant that is in defective quorum sensing and shows reduced virulence in various infection models (Salunkhe et al. 2005; Carter et al. 2010) and (3) LES431, an isolate associated with the infection of the non-CF parent of a CF patient, an upregulated quorum sensing system and enhanced virulence in infection models (McCallum et al. 2002; Salunkhe et al. 2005; Carter et al. 2010). All three LES isolates are methionine auxotrophs.

In addition, we include a genome-sequenced representative of the other most commonly studied U.K. epidemic strain, the Manchester strain, C3719 (Mathee et al. 2008; Jones et al. 2010). A number of transmissible strains have been reported in Australia, and we include representatives of the three most widely studied, namely AUST-01 (AES-1), AUST-02 (AES-2) and AUST-03 (AES-3). AUST-01, associated with increased patient morbidity, was first reported in Melbourne but has also been reported in Sydney and Brisbane (Armstrong et al. 2003; O'Carroll et al. 2004; Kidd et al. 2013). AUST-02 has been reported as more common in Brisbane (Syrmis et al. 2004; Kidd et al. 2013), whereas AUST-03 is the most common CF strain in Tasmania (Bradbury et al. 2008). Strain DK2, identified as infecting multiple CF patients in Denmark, was the subject of a detailed analysis of genome sequences from multiple isolates (Yang et al. 2011a). Although we recognize that there are other known CF transmissible strains (Fothergill et al. 2012a), and new strains are emerging all the time, we have restricted our choices to those for which there are substantial additional phenotypic and genotypic data.

#### Other CF isolates

In choosing our CF isolates (epidemic and nonepidemic strains) we sought to represent phenotypes typically associated with such isolates, such as mucoidy, hypervirulence, loss of virulence activities (such as quorum sensing), antimicrobial resistance, and auxotrophy.

Adaptation is a hallmark of CF pathogens, enabling them to chronically colonize the challenging host environment and avoid immune detection during chronic colonization (Callaghan and McClean 2012). There have been a number of studies where single-strain sequential isolates for CF patients have been analyzed in order to understand the mechanisms of the adaptation that *P. aeruginosa* populations undergo during chronic lung infections (Smith et al. 2006; Bragonzi et al. 2009; Cramer et al. 2011; Yang et al. 2011b). Classically, these include examples of isolates from “early” and “late” in an infection, and we have included example isolates from both European and North American studies. We have included isolates AA2 (early), AA43 (mucoid, late) and AA44 (nonmucoid, late), which have been compared using multiple phenotypic tests and in a murine infection model (Bragonzi et al. 2009). We also include five pediatric sequential isolates including the matched “early” and “late” isolates AMT 0023-30 and AMT 0023-34, the latter of which is an example of a hypermutator (*mutS* mutant) and shows a 100-fold increase in persister levels (enhanced survival upon exposure to antibiotics) (Mulcahy et al. 2010). We further include AMT0060-1, -2 and -3, “early” and “late” isolates from a separate patient, representing sequential isolates obtained when that patient was 15.4 (two distinct phenotypes, AMT0060-1 and -2) and 7.7 (AMT0060-3) years-old.

#### Widely studied strains

We have included in the panel a number of strains that are frequently studied by the *P. aeruginosa* research community. We note that there are variants (Klockgether et al. 2010) of the most widely studied strain, PAO1, which was the first to be genome sequenced (Stover et al. 2000). This can lead to difficulties when comparisons are made between laboratories. The variant of PAO1 we have deposited is derived from the culture grown for genome sequence analysis (Stover et al. 2000). The Pseudomonas Genome Database (http://v2.pseudomonas.com) (Winsor et al. 2011) represents a very useful and comprehensive online database for interrogation of the genome of strain PAO1, as well as the genomes of other strains included in this panel (2192, 39016, C3719, DK2, LES B58, and UCBPP-PA14) (Table 2).

It has been shown that PA14-like strains and Clone C are the two most abundant *P. aeruginosa* clones among CF patients (Romling et al. 2005; Cramer et al. 2011, 2012). Indeed, Clone C is ubiquitous throughout the inanimate environment also, whereas UCBPP-PA14 (PA14) is not so common outside of the CF population (Pirnay et al. 2009). Hence, representatives of both of these clones have been included. Strain PAK has also been widely studied, for example in relation to biofilm formation (Vasseur et al. 2005), flagellar glycosylation (Miller et al. 2008), and gene regulation (Brencic and Lory 2009).

We have also included the highly pathogenic strains CHA (Toussaint et al. 1993) and TBCF10839 (Tummler et al. 1991; Klockgether et al. 2013). Strain CHA is an example of a strain producing the potent exotoxin U, and its type III secretion system has been the subject of much analysis (Dacheux et al. 2000; Ader et al. 2005). The virulence of strain TBCF10839 has also been extensively studied (Bohn et al. 2009).

#### Strains with specific phenotypic characteristics

Mucoidy is a phenotype associated with chronic colonization, which derives from the production of high concentrations of the exopolysaccharide, alginate. Mucoid strains that chronically colonize the lungs of CF patients may evolve from initial nonmucoid strains, but the mucoid phenotype is unstable in vitro. Consequently, the pair of strains IST27, a mucoid isolate from a CF patient, and its nonmucoid variant IST27N, obtained spontaneously during IST27 cultivation in the laboratory, have been included to directly assess the role of mucoidy in pathogenesis and the regulation of the mucoid phenotype switch. IST27 and IST27N are clonal variants indistinguishable by genomic fingerprinting (Leitao et al. 1996). In contrast to IST27, IST27N has undetectable levels of GDP (guanosine diphosphate)-mannose dehydrogenase (GMD) activity, consistent with the concept that the control of alginate biosynthesis occurs at the level of the encoding gene *algD* (Tavares et al. 1999). In addition, other mucoid strains such as NH57388A and 968333S have been included (manuscript under review). The latter is a mucoid strain and was selected due to its isolation from a U.K. patient with advanced non-CF bronchiectasis treated with long-term colistin. This will provide a useful comparator to “late phase” CF strains.

Lipopolysaccharide (LPS) composition is another important phenotype that plays a key role in pathogenesis; therefore isolates with defined LPS structures have been included (Cigana et al. 2009). Furthermore, strains 39,106 (Stewart et al. 2011) and 679 were chosen as examples of severe keratitis and urinary tract infection isolates, respectively.

#### Strains representing genotypic, geographic and source diversity

Although conscious of keeping the reference panel to a reasonable number, we included a number of strains to ensure that we captured the diversity of *P. aeruginosa* in nature as much as possible. There have been a number of key studies using genotypic and phenotypic approaches to defining the population structure (Pirnay et al. 2002, 2009; Wiehlmann et al. 2007; Cramer et al. 2012; Kidd et al. 2012; Shankar et al. 2012; Martin et al. 2013). We selected seven isolates from a *P. aeruginosa* study (Pirnay et al. 2009) representing diversity in serotype, drug resistance, and geographic source, including three serotype 1 strains from very different geographic locations, and environmental isolates. Cramer et al. (2012) recently defined the population structure of *P. aeruginosa* in relation to CF by AT genotyping a collection of 955 isolates from multiple European CF centers. The collection also included isolates from various non-CF clinical and environmental sources for comparison. We have included in our collection representatives of each of the 10 most common clones identified. We have also ensured that we have representatives from diverse types of infection, including various non-CF respiratory infections (pneumonia, including ventilator-associated pneumonia (VAP), chronic obstructive pulmonary disease (COPD)-associated, non-CF bronchiectasis), burn wound infections, eye infections, and urinary tract infections. Although we accept that other genotyping approaches have been used to study large collections of *P. aeruginosa*, including multilocus sequence typing (MLST) and variable number of typing repeats (VNTR), and that all such methods have limitations, using the AT method we were able to easily place the panel strains in the context of a much wider survey and ensure that common clones are represented. It is likely that all such genotyping methods will be superceded by whole genome sequencing. Hence, we are committed to ensuring that each panel strain is genome sequenced. This is currently being undertaken in collaboration with Roger Levesque (Université Laval) and the data will be made available as soon as possible.

## Discussion

There is a need to coordinate our collective efforts in *P. aeruginosa* research to achieve both rapid and meaningful progress. Arguably the most pressing need for accelerating scientific progress relates to CF due to the clinical burden and prognostic effects of *P. aeruginosa* infections. Hence, our efforts were focussed on defining a core reference panel of isolates relevant to CF research. However, given the increasing importance of *P. aeruginosa* in a range of opportunistic infections, we have also chosen a panel with broader relevance.

We realize that no panel will achieve absolute consensus on all of the required parameters that various international research teams may wish to study. However, the consensus-seeking process did define core characteristics necessary for the proposed reference panel of *P. aeruginosa*. These characteristics included ensuring diversity in the biologic niche by choosing clinical, environmental, and laboratory isolates. We have also ensured that geographic diversity is reflected, and that although the panel includes representatives of the dominant circulating *P. aeruginosa* clones, we also include more unusual outliers to reflect the diversity of the species. Hence, we believe that within the limitations of keeping the number manageable, we have assembled a representative panel for use by the wider research community.

Infections of the CF lung by *P. aeruginosa* have been associated with the development of a number of important bacterial characteristics such as induction of mucoid status (Govan and Deretic 1996), evidence of hypermutability (Oliver et al. 2000), changes in cell surface virulence determinants (Cigana et al. 2009), and loss of virulence factors (D'Argenio et al. 2007). However, it is important to note that during chronic infections of the CF lung *P. aeruginosa* populations are diverse. Hence, there is considerable variability in virulence factor expression or antibiotic resistance among CF isolates of *P. aeruginosa,* even when they are obtained from the same sputum sample and the patient is infected with a single strain (Foweraker et al. 2005; Mowat et al. 2011). These features of *P. aeruginosa* associated with CF may be due to the (passive) accumulation or (active) development of mutations that occurs during the chronic lung infections that characterize CF. Furthermore, there are data demonstrating “CF specific” differences in the expression of virulence determinants such as LPS as compared to isolates from other clinical diseases states and environmental isolates (Ernst et al. 2007; Moskowitz and Ernst 2010). We have sought to represent in the panel some of the key phenotypic variations evident in the wider *P. aeruginosa* community, especially in relation to CF infections.

We achieved consensus on the requirement that the panel must include CF epidemic strains (Fothergill et al. 2012a) and sporadic isolates responsible for clinical infections. We also achieved consensus on the need for multiple isolates of certain important strains (e.g., LES), including subtypes with different virulence characteristics.

Antimicrobial resistance and biofilm information are both characteristics central to the clinical challenges in managing CF and other infections. The panel therefore includes multidrug-resistant strains and an isogenic parent and mutant strain which are, respectively, biofilm-forming and biofilm-deficient. It is the intention that such a panel should be used for further in-depth analysis and comparisons of phenotypes such as biofilm formation and antimicrobial resistance, and there was agreement that this requires further study. It is also envisaged that the panel will represent an excellent strain reference set for the testing of novel therapeutic approaches to the treatment of *P. aeruginosa* infections, which are desperately needed (Fothergill et al. 2012b).

Making use of prior data is scientifically and economically mandatory and improves the effective use of research funding. The proposed panel has aimed to include isolates that had already been well-characterized, such as the commonly used strains PAO1, PA14, PAK, and LESB58, and isolates where there has been comprehensive pathogenicity work undertaken.

Importantly, there was consensus that all of the identified factors did not need to be defined for each strain in the panel at inclusion. Full genome sequencing was not felt mandatory for inclusion in the panel. However, the majority of strains included have been sequenced and the remainder will be sequenced in the near future for completion. Furthermore, the number of strains included is beyond the consensus achieved for an “ideal” number. It proved difficult to reduce the number of strains included without losing the richness of the existing data already available on many of these strains.

Significant challenges lie ahead in understanding the biology of *P. aeruginosa*. The coordination of scientific efforts across research groups and avoiding the use of widely differing random isolates which result in unhelpful repetition is imperative. Better coordination and definitive replicate data fulfil an unwritten demand of the scientific community, the patient population and life science research funders. The aim of this proposed panel is to harmonize and coordinate the ongoing efforts of the research community to fulfil these goals. The international reference panel of *B. cepacia* complex isolates undoubtedly led to more streamlined approaches with this less prevalent group of pathogens. The Bcc reference panel grew with time to reflect the needs of the bioscience community, as well as to mediate the discovery of newer species within the complex being defined. This proposed panel of *P. aeruginosa* isolates has a particular focus on CF and human disease. As occurred with the original Bcc reference panel, which comprised 30 strains originally (Mahenthiralingam et al. 2000), but was updated with time and new discoveries (Coenye et al. 2003), this panel may similarly need to be extended in time. However, it is clear that a focussed panel of *P. aeruginosa* isolates as assembled is needed to help accelerate discovery and assessment of virulence determinants, and to develop better strategies to counter this successful pathogen.
